# Development of Novel Photosensitizer Using the* Buddleja officinalis* Extract for Head and Neck Cancer

**DOI:** 10.1155/2018/6917590

**Published:** 2018-06-20

**Authors:** Hyejoung Cho, Hui Zheng, Qiaochu Sun, Shuhan Shi, YuZhu He, Kyuhyeon Ahn, Byunggook Kim, Hye-Eun Kim, Okjoon Kim

**Affiliations:** ^1^Department of Dental Science, School of Dentistry, Chonnam National University, 77 Yongbong-ro, Buk-gu, Gwangju 61186, Republic of Korea; ^2^Department of Oral Maxillofacial Surgery, College of Stomatology, Dalian Medical University, Dalian 116044, China

## Abstract

Photodynamic therapy (PDT) is generally safer and less invasive than conventional strategies for head and neck cancer treatment. However, currently available photosensitizers have low selectivity for tumor cells, and the burden and side effects are so great that research is needed to develop safe photosensitizers. In this study, it was confirmed that the* Buddleja officinalis* (BO) extract, used in the treatment of inflammation and vascular diseases, shows fluorescence when activated by LED light, and, based on this, we aimed to develop a new photosensitive agent suitable for PDT. MTT, Diff-Quick® staining, and DCF-DA were performed to measure the effects of treating head and neck cancer cells with BO extract and 625 nm LED light (BO-PDT). Cell cycle, TUNEL, and western blot assays, as well as acridine orange staining, were performed to explore the mechanism of BO-PDT-induced cell death. We found that when the BO extract was irradiated with 625 nm LED light, it showed sufficient fluorescence and stronger intracellular toxicity and ROS effect than the currently commercially available hematoporphyrin. BO-PDT resulted in a decrease of mTOR activity that was correlated with an increase in the levels of ATG5, beclin-1, and LC3-II, which interfere with the formation of autophagosomes. In addition, BO-PDT induced the activation of PARP and led to an increase in the expression of proapoptotic protein Bax and a decrease in the expression of the antiapoptotic protein Bcl-2. Moreover, BO-PDT has been shown to induce the autophagy pathway 4 h after treatment, while apoptosis was induced 16 h after treatment. Finally, we confirmed that BO-PDT caused cell death of head and neck cancer cells via the intrinsic pathway. Therefore, we suggest that BO extract can be used as a new photosensitizer in PDT of head and neck cancer.

## 1. Introduction

Head and neck cancer is treated using surgery, radiation therapy, and chemotherapy, either alone or in combination. However, it is not easy to cure head and neck cancer because of the complex anatomical form of the head and the neck, and it usually has severe side effects that result in substantial dysfunction and trauma [1]. Therefore, photodynamic therapy (PDT) has gained considerable interest as a noninvasive therapy for head and neck cancer [2, 3]. In PDT, a light sensitizer is activated at light of a specific wavelength and in the presence of oxygen [4]. The photosensitizer may react to light alone or when associated with cells; when combined with cells, it can be used as a suitable material for PDT if it generates fluorescence or active oxygen at light of specific wavelengths [5]. Commercial photoprotective agents currently in use have side effects and limitations when applied in patients. For example, the widely used hematoporphyrin (HP) derivative has the disadvantage that its selectivity for cancer cells is poor and its photoprotection time is considerably long. Another photosensitive agent, 5-aminolevulinic acid (5-ALA), has relatively low activity due to its relatively low light energy and long processing time. Most photosensitizers have a structure similar to porphyrin and thus similar side effects to it [6]. Therefore, it is necessary to develop new photosensitizers that are safe and have fewer side effects. In this study, as a new photosensitizer, we investigated the effect and cytological mechanism of* Buddleja officinalis* (BO) flower extract, which is reportedly effective in treating inflammation, conjunctival hypertension, and headache in traditional oriental herbal medicine [7–10].

## 2. Materials and Methods

### 2.1. Buddleja officinalis Extract

Dried* Buddleja officinalis* flowers were obtained from Dong Kyung PHARM (KYUNG DONG, Seoul, Korea). The dried herb (1,000 g) was ground to be powder with a mechanic grinder, and the powdered material weighed 300 g. The extract was then prepared with 1,500 mL of 70% EtOH at room temperature for 48 h using a dynamoelectric stirrer (MTOPS, Ankara, Turkey). After filtration with 70% EtOH extraction, it was evaporated in a rotary vacuum evaporator (EYELA, Kubota, Japan). One gram of the final product was mixed with 1 mL of 10 mM HCl. This was then diluted with ddH_2_O to 1 mg/mL (pH 6.4) and syringe-filtered.

### 2.2. Cell Lines and Cell Culture

FaDu head and neck squamous cell carcinoma cells were purchased from the Korean Cell Line Bank (Seoul, Korea) and cultured in Dulbecco's modified Eagle's medium (DMEM; Gibco BRL, Gel Company, San Francisco, CA, USA) supplemented with 10% fetal bovine serum (FBS; JR Scientific Inc., Woodland, CA, USA) and penicillin/streptomycin (Gel Company) at 37°C in a humidified atmosphere of 5% CO_2_.

### 2.3. Light Irradiation Conditions

**Figure 1:**
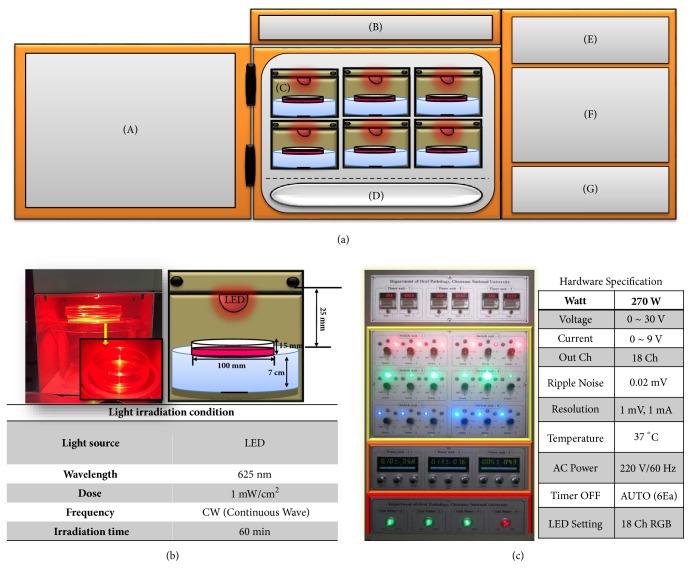
**Schematic of LED irradiation machinery and tools**. LED irradiation machinery and tools for in vitro testing were built in a CO_2_-humidified chamber. (a) LED irradiation machinery and tools consist of CO_2_ incubator and controller: (A) CO_2_ incubator door, (B) incubator display window, (C) 625 nm LED light source, (D) water tank, (E) timer, (F) wavelength adjuster, (G) main power switch, and (b) LED investigation of cell culture dishes. For a uniform light energy distribution, the distance between the cell culture medium and the test board was adjusted to 25 mm, so that the LED was designed to illuminate the entire bottom of the Petri dish. The top view of cell culture dish clearly shows high transparency of cell culture containers to LED light. (c) Hardware control system. The controller sets the incubation time and fluorescence. The blue part is the timer part which turns the output signal on/off. The yellow part is the adjuster for adjusting the wavelength of the LED, the orange part is the power meter, and the red part is the main power switch. The total wattage is 270 W, the voltage variable is 0 ~ 30 V, the current variable is 0 ~ 9 A, the output channel for total experiment is 18 channels, and 18 experiments are designed to be carried out simultaneously.

As a light source, a continuous-wave LED (U-JIN LED, Goyang, Korea), emitting light at a wavelength of 625 nm, at a power density of about 1 mW/cm^2^, on a sample surface at a temperature of 37°C in a humidified atmosphere of 5% CO_2_, was used. Each experimental group was irradiated for 1 h (Figure 1).

### 2.4. Fluorescence Emitting Analysis

To confirm the possibility of BO extract being used as a natural photosensitizer, we measured and compared the fluorescence levels of the BO extract and HP, a commonly used photosensitizer. The BO extract and HP (Sigma-Aldrich, St. Louis, MO, USA) were diluted to 1–500 *μ*g/mL with dimethyl sulfoxide (DMSO; Calbiochem, Burlington, MA, USA). Fluorescence was measured using an FL X800 Microplate Fluorescence Reader (excitation, 420/50 nm; emission, 645/40 nm).

### 2.5. Cell Viability Assay

Cell viability was determined using an established colorimetric method using 3-(4,5-dimethylthiazol-2-yl)-2,5-diphenyltetrazolium bromide (MTT; Sigma-Aldrich). The MTT assay is based on the ability of mitochondrial dehydrogenases in viable cells to reduce MTT to formazan through cell respiration. Cells suspended in DMEM containing 10% FBS were transferred into a flat-bottomed 96-well plate (Corning Inc., Corning, NY, USA) at a density of 1 × 10^5^ cells/mL. The cells were subsequently treated with 40 *μ*g/mL of BO extract for 24 h and then irradiated with 625 nm LED light for 1 h. The medium was removed 24 h after the experimental treatments, and the cells were then incubated in phosphate-buffered saline (PBS) containing 30 *μ*L of MTT at 37°C for 3 h. The formazan product was solubilized by the addition of 50 *μ*L of DMSO. The optical density was measured at 570 nm using an enzyme-linked immunosorbent assay reader (ELx800uv; BioTek Instruments Inc., Winooski, VT, USA).

### 2.6. Cell Cycle Analysis

Cultured cells were trypsinized and fixed with 70% EtOH at 4°C overnight, followed by staining with propidium iodide (PI; Sigma-Aldrich). DNA content was analyzed by flow cytometry (FACS; Beckman-Coulter, Brea, CA, USA).

### 2.7. Determination of the Intracellular Localization of BO Extract

To examine the intracellular localization of the BO extract, we monitored the photosensitizer using confocal microscopy (Carl Zeiss, Oberkochen, Germany). Cells were grown on cover slides and incubated in DMEM containing 40 *μ*g/mL of the BO extract for 1, 2, 4, and 8 h. After incubation, the cells were treated using 4% formaldehyde fixative solution (T&I Co., Ltd., Gwangju, Korea) for 5 min, washed twice with PBS, and then mounted onto slides. Finally, the cells were examined by confocal microscopy at excitation and emission wavelengths of 530–560 and 590–650 nm, respectively.

### 2.8. Detection of Cellular Reactive Oxygen Species (ROS)

To measure intracellular ROS levels, a fluorescent probe, 2′,7′-dichlorodihydrofluorescein diacetate (DCF-DA; Sigma-Aldrich), was used. Cells were grown on cover slides and incubated in DMEM containing 40 *μ*g/mL of BO extract or HP for 24 h. After irradiation with 625 nm LED light for 1 h, the cells were stained with 25 *μ*M DCF-DA, washed twice with PBS, and mounted on slides. Finally, the cells were examined under a confocal microscope, at 488 nm excitation and 530 nm emission filters.

### 2.9. Diff-Quick Staining

To analyze morphological changes of FaDu cells, cells were seeded on cover slips in 6-well plates (Corning Inc.) at a density of 1 × 10^5^ cells/mL and treated with 40 *μ*g/mL of BO extract. After 24 h, they were irradiated with 625 nm LED light for 1 h. The cells were washed with PBS and fixed with 4% paraformaldehyde for 20 min at room temperature. The fixed cells were treated with Diff-Quick solution (Sysmex Co., Kobe, Japan). Briefly, the cells were stained with Diff-Quick solution II for 30 s, washed with PBS, counterstained with Diff-Quick solution I for 30 s, rinsed in tap water to remove excess stain, and rapidly dehydrated in absolute alcohol. Cell morphology was examined by optical microscopy (Olympus, Tokyo, Japan).

### 2.10. Detection of Apoptosis by TUNEL Assay

To observe DNA strand breaks in the nuclei at the cellular level, a terminal deoxynucleotidyl transferase-mediated dUTP nick end-labeling (TUNEL) assay was performed using a TUNEL Assay System (R&D Systems, Minneapolis, MN, USA) according to the manufacturer's directions. Briefly, the cells were fixed with 4% paraformaldehyde and then washed with PBS at room temperature. The cells were permeabilized with Triton® X-100 (Sigma-Aldrich) and washed with PBS. The incubation buffer, containing equilibration buffer, nucleotide mix, and rTdT enzyme, was added and the cells were incubated at 37°C for 1 h. Afterwards, nuclear staining with DAPI was performed. Finally, the cells were observed under a confocal microscope. Localized green fluorescence of apoptotic cells (fluorescein-12-dUTP) on a blue DAPI background was detected by confocal microscopy.

### 2.11. Acridine Orange Staining

To detect whether BO extract induced autophagic cell death, we treated cells with 40 *μ*g/mL of BO extract for 24 h. The cells were then briefly washed using PBS and incubated with 0.5 *μ*g/mL acridine orange hydrochloride solution (Molecular Probes, Eugene, OR, USA) in PBS for 20 min at room temperature and afterwards analyzed by confocal microscopy. The cytoplasm and nuclei of the stained cells exhibited bright green fluorescence and acidic autophagic vacuoles exhibited bright red fluorescence.

### 2.12. Western Blot Analysis

Whole cells were lysed using RIPA buffer, containing 50 mM Tris-HCl (pH 7.5), 150 mM NaCl, 1 mM EDTA, 1% NP-40, 1 mM phenylmethanesulfonyl fluoride, and 1% protease inhibitor cocktail. The proteins were then separated by 10% sodium dodecyl sulfate polyacrylamide gel electrophoresis and detected using primary antibodies. The following protein markers related to the apoptosis pathway were used: anti-PARP-1 (1:1000), anti-Bax (1:1000), and anti-Bcl-2 (1:1000); the following protein markers related to the autophagy pathway were used: anti-ATG-5 (1:1000), anti-beclin-1 (1:1000), anti-LC3-II (1:1000), anti-pmTOR (1:1000), and anti-mTOR (1:1000). To further examine the intracellular effects of BO extract and 625 nm LED irradiation and to investigate the affected pathway, anti-pAMPK (1:1000), anti-AMPK (1:1000), anti-pAKT (1:1000), anti-AKT (1:1000), and anti-GAPDH (1:2000) were used (Cell Signaling Technology, Inc., Danvers, MA, USA). Anti-rabbit IgG (sc-2004) and anti-mouse IgG (sc-2005) were used as secondary antibodies. The immunoreactivities of the proteins were detected with an enhanced chemiluminescence detection kit (GE Healthcare, Little Chalfont, UK).

### 2.13. Caspase-3, Caspase-8, and Caspase-9 Activity Assays

Caspase-like protease activity (caspase-3, caspase-8, and caspase-9) was measured using ApoAlert™ apoptosis assay kits (Clontech, USA). Briefly, cultured cells were lysed in cell lysis buffer, containing 20 mM Tris-HCl (pH 7.5), 150 mM NaCl, 1 mM disodium EDTA, 1 mM EGTA, 1% Triton, 20 mM sodium pyrophosphate, 25 mM sodium fluoride, 1 mM *β*-glycerophosphate, 1 mM Na_3_VO_4_, and 1 *μ*g/mL leupeptin. The cell lysates were incubated with specific colorimetric peptide substrates for each caspase and a reaction buffer containing 100 mM 1, 4-dithiothreitol. The caspase-3 and caspase-8 activity assays were performed using a microplate reader at 405 nm, while the caspase-9 assay was performed using 380 nm excitation and 460 nm emission.

### 2.14. Statistical Analysis

Data are expressed as mean values ± standard deviations. All experiments were repeated three times and one-way ANOVA test followed by the post hoc statistical test was used to evaluate the differences between groups. The difference was considered statistically significant if* P* < 0.05.

## 3. Results

### 3.1. Effects of BO on Fluorescence Levels and Cell Viability in FaDu Cells

**Figure 2:**
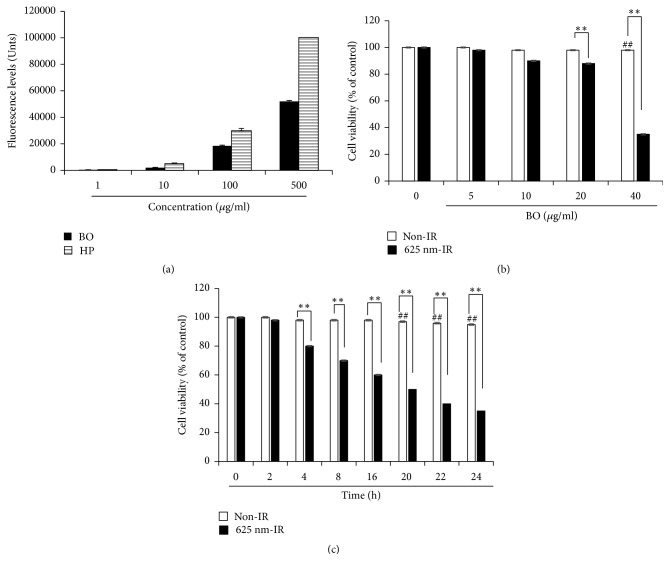
**BO-PDT-induced cell death in FaDu cells**. (a) The fluorescence values of BO extract and hematoporphyrin (HP) at 1, 10, 100, and 500 *μ*g/mL. The maximum value that could be measured by a microplate fluorescence reader was represented as 100,000 units. The cytotoxicity of FaDu cell treatment with BO extract and 625 nm LED light (BO-PDT). (b) The effects of BO-PDT at BO extract concentrations of 5, 10, 20, and 40 *μ*g/mL are shown. (c) BO-PDT decreased cell viability in a time-dependent manner. Nontreated condition versus BO: #* P* < 0.05; ##* P* < 0.001; BO versus BO-PDT: *∗P *< 0.05; *∗∗P* < 0.01.

The fluorescence levels of HP and BO extract were compared. The fluorescence levels of HP and BO extract increased in a dose-dependent manner. HP reached its maximal fluorescence value at a concentration of 500 *μ*g/mL, and BO extract did not reach the fluorescence levels of HP. As shown in Figure 2(b), FaDu cells, originating from oral cancer, were treated with BO extracts at concentrations of 5–40 *μ*g/mL to determine cell viability. BO extract without 625 nm LED irradiation did not show cytotoxicity. However, when cells were treated with BO extract and irradiated with 625 nm LED irradiation (BO-PDT), the treatment showed cytotoxicity and decreased cell viability in a dose-dependent manner. In particular, at a concentration of 40 *μ*g/mL, the survival rate of the cells was decreased by less than a half when compared to that of the control. In addition, when the cell survival rate using the BO extract concentration of 40 *μ*g/mL was assessed over time, the BO extract without 625 nm LED irradiation did not show cytotoxicity. However, when the cells were subjected to BO-PDT, the survival rate of the cells decreased over time, starting from 4 h after BO-PDT (Figure 2(c)).

### 3.2. Effects of BO Extract on Cell Morphology and ROS Levels in FaDu Cells

**Figure 3:**
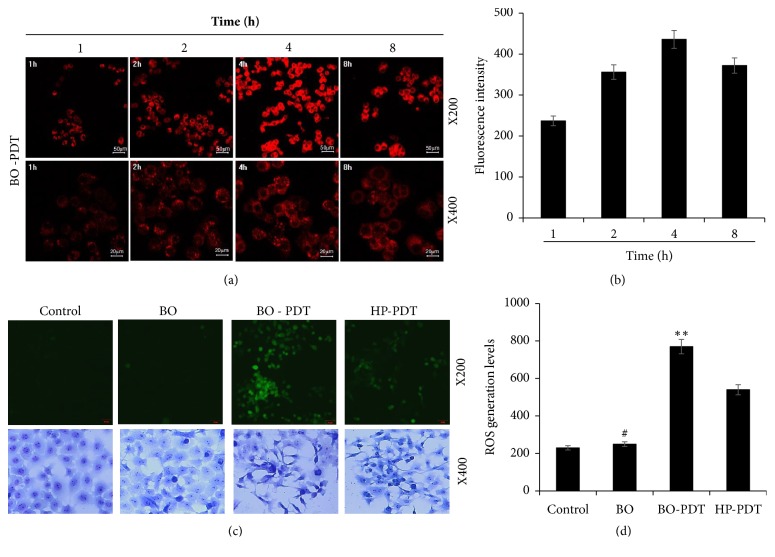
Determination of intracellular localization of BO extract and ROS generation and morphological changes induced by treatment with BO extract and 625 nm LED irradiation. (a) Photomicrographs showing the intracellular fluorescence of BO at various incubation times in the FaDu cell line. Confocal microscopy revealed that BO-PDT was localized in the cell membrane and the cytoplasm, excluding the cell nucleus. The fluorescence intensity in stained cells increased during 1–4 h. (b) The fluorescence intensity of BO extract evaluated with Image J. (c) DCF-DA assay, performed to compare ROS levels in FaDu cells treated with BO extract and those treated with hematoporphyrin (HP) (upper panel). The ROS levels after BO-PDT were significantly higher than after HP-PDT. The cells were subsequently stained with Diff-Quick staining (Lower panel) and examined for morphological changes after BO-PDT. The red arrow indicates cell undergoing apoptosis. (d) The fluorescence intensity of DCF-DA in the cells evaluated with Image J. Nontreated condition versus BO: #* P* < 0.05; ##* P* < 0.001; BO versus BO-PDT: *∗P *< 0.05; *∗∗P *< 0.01.

We assessed whether the localization of fluorescence in BO-treated cells changed over time. The fluorescence was first very intense in the cell membrane and in parts of the cytoplasm, but not in the nucleus. In addition, the strongest fluorescence was observed at 4 h after treatment with BO extract, and the fluorescence gradually faded (Figure 3(a)). Furthermore, we compared intracellular ROS levels after treatment with BO extract or HP by the DCF-DA assay. The ROS levels after treatment with 40 *μ*g/mL of BO extract were slightly higher than those in the untreated control group, and the ROS levels in cells after BO-PDT were significantly higher than those after treatment with HP and 625 nm irradiation (HP-PDT) (Figure 3(b), upper panels). In addition, we observed changes in cell morphology after BO-PDT using the Diff-Quick stain. The cell shape and cell number did not change after treatment with BO extract alone compared to that of the untreated control. However, after BO-PDT, the cell number was reduced and the cell shape markedly changed from round to elongated (Figure 3(b), lower panels).

### 3.3. Effect of BO-PDT on the Apoptotic Pathway in FaDu Cells

**Figure 4:**
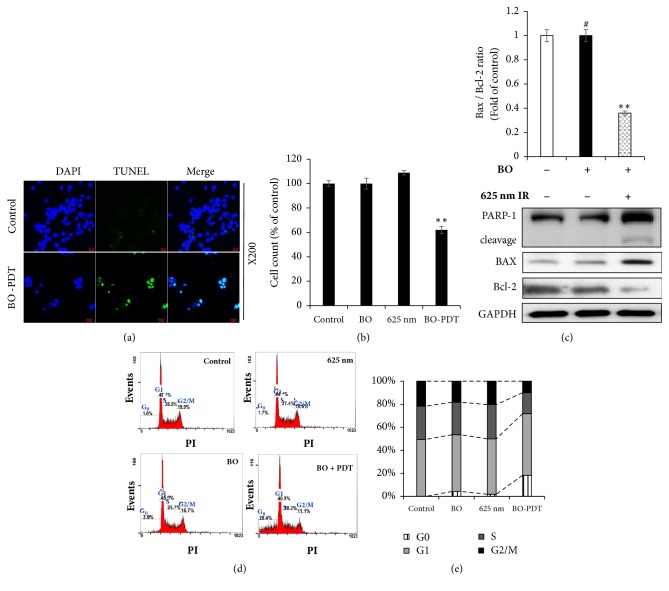
**Effects of BO-PDT on apoptosis**. (a) The effect of BO-PDT on apoptosis in FaDu cells was evaluated by TUNEL assay. Representative images of TUNEL assay in FaDu cells (magnification ×200). (b) The cells were quantified using DAPI staining. (c) Effects of BO-PDT on expression of apoptotic proteins in FaDu cells. Western blot analysis of the expression of PARP-1, cleaved PARP-1, Bcl-2, and Bax. Equal protein loading was confirmed by the antibody reaction to GAPDH. (d) Effects of BO-PDT on the cell cycle of FaDu cells. DNA content was analyzed by PI staining (G0, G1, S, and G2/M). (e) Cell cycle results are graphed. Nontreated condition versus BO: #* P* < 0.05; ##* P* < 0.001; BO versus BO-PDT: *∗P *< 0.05; *∗∗P *< 0.01.

When FaDu cells were subjected to BO-PDT, the ratio of TUNEL-positive apoptotic cells increased compared with the control group (Figures 4(a) and 4(b)). To investigate the mechanism of apoptosis induced by BO-PDT, the expression levels of PARP-1, cleaved PARP-1, Bax, and GAPDH were measured in FaDu cells 24 h after BO-PDT. Levels of the proapoptotic protein Bax were significantly higher, while levels of the antiapoptotic protein Bcl-2 were lower after BO-PDT. Treatment with BO only did not significantly affect the levels of Bax or Bcl-2 compared to the untreated control (Figure 4(c)). Cell cycle analysis showed that there was no significant difference between the groups treated with BO extract (sub-G0: 3.9%) and 625 nm LED irradiation (sub-G0: 1.7%) alone compared to the untreated control (sub-G0: 1.6%). However, after BO-PDT, the sub-G0 population (sub-G0: 20.4%), which indicates apoptotic cell death, significantly increased (Figures 4(d) and 4(e)).

### 3.4. Effect of BO-PDT on the Autophagy Pathway in FaDu Cells

**Figure 5:**
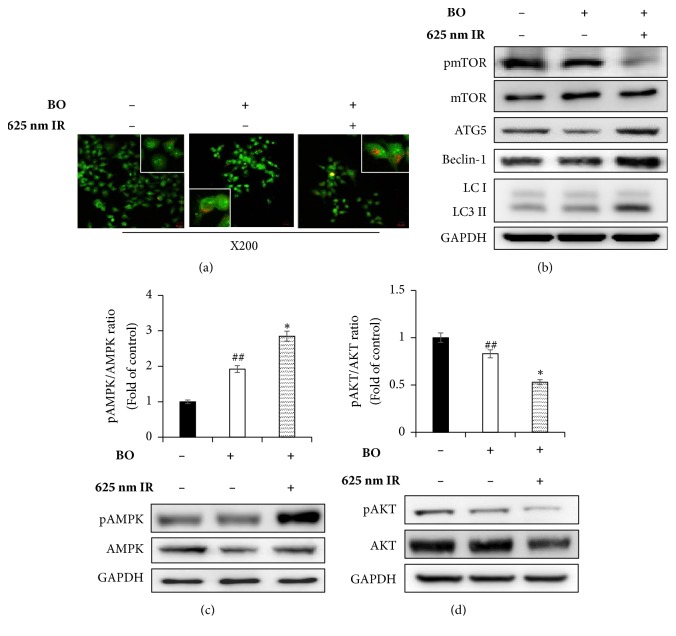
**Effects of BO-PDT on autophagy**. Acidic vesicular organelle (AVO) formation was detected using acridine orange staining. (a) Microscopic photos taken at ×100 magnification. (b) The cells were subjected to western blot to measure pmTOR, mTOR, ATG5, beclin-1, and LC3-II levels. (c, d) Western blot, performed to measure the expression levels of pAMPK, AMPK, pAKT, and AKT. Equal protein loading was confirmed by the antibody reaction to GAPDH. Protein levels were measured by densitometry, and their relative levels were compared. Nontreated condition versus BO: #* P* < 0.05; ##* P* < 0.001; BO versus BO-PDT: *∗P* < 0.05; *∗∗P* < 0.01.

After 24 h of incubation with BO extract or after BO-PDT, acridine orange staining was performed. The majority of cells exhibited green fluorescence in the untreated control and the BO extract-treated groups. Cells only irradiated with LED showed results similar to those of the untreated control. The cells subjected to BO-PDT developed orange and orange-red fluorescence, indicating membrane disruption (Figure 5(a)). In addition, mTOR protein levels decreased, while ATG5, beclin-1, and LC3-II protein levels increased after BO-PDT (Figure 5(b)). Both an increase in pAMPK and a decrease in pAKT levels were observed, while there was no significant difference between the untreated control and the BO extract-treated groups (Figures 5(c) and 5(d)).

### 3.5. BO-PDT Induces the Autophagic Pathway before the Intrinsic Apoptosis Pathway

**Figure 6:**
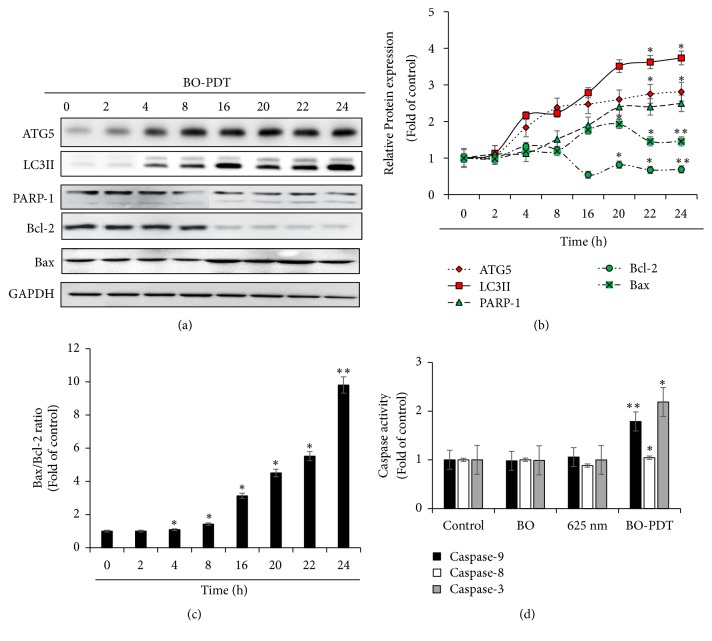
**Effects of BO-PDT on cell death mechanism**. (a) The protein levels of ATG5, LC3-II, PARP-1, Bax, and Bcl-2 over time. Equal protein loading was confirmed by the antibody reaction to GAPDH. (b) Summary of the effects of BO-PDT on the expression of ATG5, LC3-II, PARP-1, Bcl-2, and Bas over time. Protein levels shown in (a) were measured by densitometry, and their relative levels were compared with the untreated control. (c) Bax/Bcl-2 protein ratio was quantified. *∗ P* < 0.05 and *∗∗ P* < 0.01 versus nontreated control (0 h). (d) Caspase-3, caspase-8, and caspase-9 activity was measured by colorimetric protease assays.

To determine the time of autophagy and apoptosis onset, we examined the expression levels of autophagy and apoptosis proteins over time. As shown in Figure 6, the expression levels of ATG5 and LC3-II, which are associated with autophagy, increased at 4 h after BO-PDT. However, the expression levels of PARP-1 and Bax, which are associated with apoptosis, increased at 16 h after BO-PDT. Moreover, the expression levels of Bcl-2, a known inhibitor of both of autophagy and apoptosis, decreased at 4 h after BO-PDT. To determine the detailed mechanism of apoptosis in cells subjected to BO-PDT, we investigated the activity of caspase-3, caspase-8, and caspase-9 at 16 h after BO-PDT, when an increase in the expression of apoptosis-related proteins was observed. BO-PDT induced significant activation of caspase-9 (1.7 ± 0.43-fold), the initiator of the intrinsic apoptosis pathway, and of caspase-3 (2.2 ± 0.65-fold). The activity of caspase-8, the initiator of the extrinsic apoptosis pathway, did not increase at this timepoint (Figure 6(c)).

## 4. Discussion

In the present study, we demonstrated that BO extract could be used as a photosensitizer, coupled with 625 nm LED irradiation, which induces cell death in the head and neck cancer cell line FaDu, and we investigated the molecular mechanism by which BO-PDT induces apoptosis and autophagy. BO extract itself showed weak fluorescence compared to HP (Figure 2(a)); however, it showed stronger ROS generation ability than HP during PDT in FaDu cells (Figure 3(b)). The accumulation of ROS-damaged cytoplasmic components is the main mechanism of PDT [11]. Moreover, photosensitizer uptake by cancer cells is crucial for activating PDT. Photosensitizers trigger cell damage induced by ROS in the proximity of key organelles such as the nucleus, plasma membrane, and mitochondria. ROS have a short half-life and can only act at the site of generation. Therefore, the effect of the photosensitizer depends on its precise subcellular localization [12]. During PDT, cell membranes, which contain a variety of signaling molecules and receptors, are destroyed, which can have various effects in cancer treatment [13]. As shown in Figure 3(a), BO extract showed sufficient fluorescence intensity on the cell membrane and in parts of the cytoplasm in FaDu cells, and its localization and fluorescence intensity were sustained for over 4 h. For successful clinical BO-PDT, it is very important to understand the localization and cellular uptake mechanisms of the BO extract to develop an effective molecule that will facilitate the entry of BO extract into specific cells. Further studies are required to confirm the exact localization of the BO extract using fluorescent organelle-specific dyes. In addition, BO extract treatment alone was not cytotoxic; however, in BO-PDT, the survival rate of cells dramatically decreased (Figures 2(b) and 2(c)). These results suggest that BO extract can be used as a photosensitizer that is activated at the appropriate wavelength and energy and that does not produce toxic metabolites during PDT. Moreover, the safety of treating cells with BO extract is higher than treating them with HP. In addition, BO extract shows greater ROS production ability in PDT than HP. Further, BO-PDT induced the activation of PARP, increased the expression of the proapoptotic protein Bax, and decreased the expression of the antiapoptotic protein Bcl-2 (Figure 4). Figure 4(c) shows a change in the ratio of Bax to Bcl-2, which contributed to the proapoptotic effect of BO-PDT. Moreover, our results show that BO-PDT decreased mTOR activity, which was correlated with an increase in the levels of ATG5, beclin-1, and LC3-II, all of which interfere with the formation of autophagosomes (Figure 5(b)). The AKT/mTOR signaling pathway is important in regulating autophagy: suppression of AKT decreases mTOR activity and promotes autophagy [14]. Meanwhile, AMPK-mediated mTOR inhibition also promotes autophagy [15]. As shown in Figure 5, 24 h after FaDu cells were subjected to BO-PDT, acridine orange staining was performed: the majority of untreated cells and cells treated with BO only exhibited green fluorescence. Cells only subjected to LED irradiation showed results similar to the untreated control, while cells subjected to BO-PDT developed orange and orange-red fluorescence, indicating membrane disruption (Figure 5(a)). In addition, mTOR protein levels decreased, and ATG5, beclin-1, and LC3-II protein levels increased (Figure 5(b)). Both an increase in pAMPK and a decrease in pAKT were observed, while there was no significant difference between the untreated control group and the group treated with BO extract only (Figures 5(c) and 5(d)). The phosphorylation of AKT was decreased, and the phosphorylation of AMPK was increased. These results suggest that BO-PDT induced autophagy in FaDu cells via the inhibition of mTOR, which requires AKT inhibition and AMPK activation. In this study, we demonstrated that BO extract induces high ROS generation and cell death via both apoptosis and autophagy upon irradiation with 625 nm LED light. It is well known that apoptosis and autophagy involve a complex cross-talk that is often induced by similar stimuli and regulated by similar pathways [16]. In order to understand the relationship between autophagy and apoptosis induced by BO-PDT in FaDu cells, we examined the expression levels of autophagy- and apoptosis-related proteins over time. Autophagy-related proteins were induced at 4 h after BO-PDT, while apoptosis-related proteins were induced at 16 h after BO-PDT (Figures 6(a) and 6(b)): in other words, apoptosis was induced later than autophagy. As shown in Figure 3(a), the strongest fluorescence was observed at 4 h after treatment with BO extract, after which the fluorescence gradually decreased (Figure 3(a)). At 4 h after treatment with BO extract, the BO extract fluorescence was first very intense in the cell membrane and in parts of the cytoplasm. Autophagy is induced as an adaptive response against endoplasmic reticulum (ER) stress, which can also contribute to ER-induced apoptosis [17]. As shown in Figure 6(d), BO-PDT led to strong activation of caspase-9, an initiator of the intrinsic pathway, after 16 h, but it did not affect the activation of caspase-8, an initiator of the extrinsic pathway. These results suggest that BO-PDT induced cell death of head and neck cancer cells via the intrinsic pathway. At 16 h after BO-PDT, the fluorescence of BO extract already faded, which may mean that prolonged activation of autophagy by BO-PDT may lead to apoptosis via the intrinsic pathway (Figure 3(a)). However, further studies are needed to elucidate the exact relationship of autophagy and apoptosis using inhibitors of apoptosis or necrosis. In conclusion, we suggest that BO extract can be used as a photosensitizer instead of the existing ones for PDT of head and neck cancer cells.

## Data Availability

The data used to support the findings of this study are available from the corresponding author upon request.
